# Optimal Control for a Parallel Hybrid Hydraulic Excavator Using Particle Swarm Optimization

**DOI:** 10.1155/2013/831564

**Published:** 2013-06-02

**Authors:** Dong-yun Wang, Chen Guan

**Affiliations:** ^1^School of Engineering, Zhejiang Normal University, Zhejiang 321005, China; ^2^School of Mechanical Engineering, Zhejiang University, Zhejiang 321005, China

## Abstract

Optimal control using particle swarm optimization (PSO) is put forward in a parallel hybrid hydraulic excavator (PHHE). A power-train mathematical model of PHHE is illustrated along with the analysis of components' parameters. Then, the optimal control problem is addressed, and PSO algorithm is introduced to deal with this nonlinear optimal problem which contains lots of inequality/equality constraints. Then, the comparisons between the optimal control and rule-based one are made, and the results show that hybrids with the optimal control would increase fuel economy. Although PSO algorithm is off-line optimization, still it would bring performance benchmark for PHHE and also help have a deep insight into hybrid excavators.

## 1. Introduction

 With the purposes of reducing fuel consumption and emissions, colleges, institutes, and companies are trying to develop a new type of excavator for the next generation. In 2003, Hitachi first produced one hybrid wheel loader which was the first hybrid construction machinery around the world. Until now, a large portion of construction machinery producers including Kobelco, Komatsu, and Caterpillar are involved in the field of hybrid excavators. 

 From the system configuration point of view, there are three types of hybrid excavators depending upon different power-train configurations. One is series hybrid excavator, another is the parallel one, and the remaining is the split one. To get detailed information regarding the properties of all these different configurations, readers would refer to the literature [1, 2]. In summary, compared with other two, the parallel configuration is considered as the one with low cost and better performance. One parallel hybrid hydraulic excavator has an additional motor that is connected to the engine via the same shaft; in addition, one storage unit (usually it is battery or capacitor) is introduced into power-train as to receive extra energy from engine to charge itself when the motor is in generation mode or deplete its energy to the pump when the motor is in motor mode. The main concepts of hybrids are to keep the engine's working points located in high fuel economy range that would badly decrease the fuel consumption regardless of working condition. Wang et al. did simulation research and evaluated energy saving effect in parallel hybrid excavators in 2005 and also presented a way to recover potential energy from excavators [2]. The parallel model, system dynamic analysis, rule-based control strategy, fuzzy logic control strategy, and component sizing are put forward by Wang et al. [3–7]. Despite of all these efforts in the field, few papers focus on the nonlinear constraints optimal control problem for PHHE.

As described in [1], the electric motor provides a fast response to the torque and speed demand with high efficiency in a large angular velocity scale. So in this paper, PSO algorithm is mainly used to get the optimal working points of the diesel engine. So the obtained group of engine's working points is globally optimized at each sampling time. This program automatically moves on to the next time interval once it finishes the previous sampling time. Thus, the global optimization for the whole cycle could be achieved. Here, PSO algorithm was first invented by Kennedy and Eberhart in 1995, that is, a stochastic global optimization approach capable of obtaining the global optimum [8]. Compared with other kinds of derivative-free algorithms such as DIRECT, simulated annealing (SA), and genetic algorithm (GA), PSO's strength lies in its simplicity, being easily coded and requiring few algorithm parameters to define convergence behavior. Also, one could use gradient-based algorithms such as sequential quadratic programming (SQP) to find the local minima, but it would not search the entire space, so the global optimum could not be obtained by this method.

 The structure of the paper is as follows.  Section 2 describes the model of the parallel hybrid hydraulic excavator and components' parameters in detail. The optimal control problem is formulated in Section 3, followed by inequality/equality constraints. PSO algorithm is then coded to deal with this specific application. The obtained set of optimum points will be implemented in the aforementioned model, and the analysis of the optimal control will be presented based on comparisons. Finally, Section 4 provides an extension on further research and a summary of this paper. 

## 2. The Plant Model

**Figure 1:**
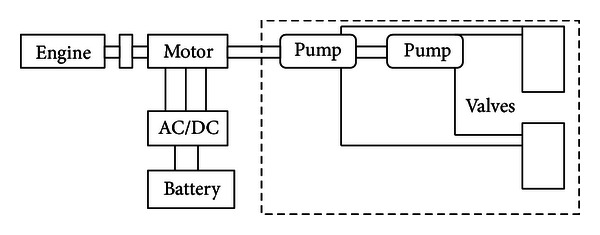
A schematic of a parallel hybrid hydraulic excavator.

 A schematic of a parallel hybrid hydraulic excavator is shown in Figure 1. The motor connects to the pump and engine together through the same shaft, and all the required power at the pumps is satisfied by the engine and motor. So with this configuration, it has three different types of modes listed as follows. (1) Motor assist hybrid mode: the engine and the motor would drive the final load together, and the motor is working under motor mode. (2) Motor generation hybrid mode: only engine delivers power to the load, and part of the power from the engine will be transmitted to the motor then will be charged into the battery. (3) Pure engine mode: only the engine works, and the motor will be rotated along with the shaft with no torque generated. The equations of the power-train are presented as follows:

(1) $$\begin{array}{c} J_{\text{eng}}\frac{\text{d}w_{\text{eng}}}{\text{d}t}=\tau_{\text{eng}\_T}-\tau_{\text{eng}},\\ J_{\text{mc}}\frac{\text{d}w_{\text{mc}}}{\text{d}t}=\tau_{\text{mc}\_T}-\tau_{\text{mc}},\\ \tau_{\text{eng}}+\tau_{\text{mc}}=\tau_{p},\\ w_{\text{eng}}=w_{\text{mc}}=w_{p},\\ P_{p\_\text{in}}=\tau_{p}w_{p}=\frac{p_{x}Q_{x}+p_{2}Q_{2}}{\eta_{p}}, \end{array}$$

where *J*_eng_, *τ*_eng_, *w*_eng_, and *τ*_eng_*T*_ are inertia, speed, torque, and total torque of the engine, respectively, and   *J*_mc_,  *w*_mc_, *τ*_mc_, and *τ*_mc_*T*_ are inertia, speed, torque, and total torque of the motor. *P*_*p*_in_, *τ*_*p*_, *w*_*p*_, *p*_*x*_, *Q*_*x*_, and *η*_*p*_ are input power, torque, speed, pressure, flow, and efficiency of the pumps. Since in current configuration of the excavator, there are 2 pumps connected in parallel by which bucket cylinder, arm cylinder, swing hydraulic motor, and boom cylinder are driven.

In addition, the dynamic equations of the battery are analyzed as follows:

(2) $$\begin{array}{c} i=\frac{U_{\text{oc}}-\sqrt{U_{\text{oc}}-2P_{\text{bat}}R_{\text{bat}}}}{2R_{\text{bat}}},\\ \text{SOC}=-\frac{U_{\text{oc}}-\sqrt{U_{\text{oc}}-2P_{\text{bat}}R_{\text{bat}}}}{2R_{\text{bat}}C_{\text{bat}}},\\ P_{\text{bat}}=\frac{\tau_{\text{mc}}w_{\text{mc}}}{\eta_{\text{mc}}^{k}}, \end{array}$$

where *U*_oc_, *P*_bat_, *R*_bat_, *C*_bat_, *i*, and SOC are open-circuit voltage, required power, inner resistor, maximum capacity, current, and state of charge of the battery, respectively. *k* indicates the motor's current mode and 1 means that the motor is under motor mode, while 0 indicates that the motor is under generation mode.

 In summary, there are several static look-up tables with which the fuel rate of the engine, open-circuit voltage of the battery, resistor of the battery, and motor's efficiencies are obtained by former experimental data:

(3) $$\begin{array}{c} {\dot{m}}_{\text{fuel}}={\dot{m}}_{\text{fuel}}\left(\text{t}{\text{h}}_{\text{eng}},\tau_{\text{eng}}\right),\\ U_{\text{oc}}=U_{\text{oc}}\left(\text{SOC},\text{temp}\right),\\ R_{\text{bat}}=R_{\text{bat}}\left(k,\text{SOC},\text{temp}\right),\\ \eta_{\text{mc}}^{}=\eta_{\text{mc}}^{}\left(k,\tau_{\text{mc}},w_{\text{mc}}\right), \end{array}$$

where ${\dot{m}}_{\text{fuel}}$ and th_eng_ are engine's fuel rate and throttle angle, and temp is the temperature value of the battery pack.

**Figure 2:**
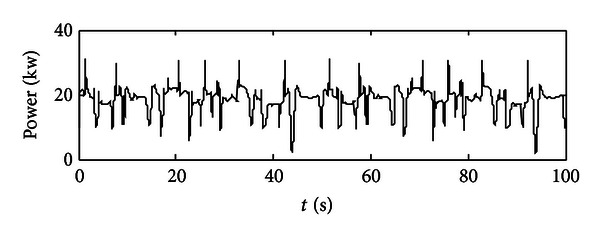
The required power at the pumps under a heavy digging cycle.

In Figure 2, the required power of the pumps is collected from experiments while the 5-ton excavator is under heavy digging condition (the rock excavation, e.g.). This power chart is considered as the required load in our model; then engine, motor, and battery would be controlled in order to meet the requirements. In Table 1, the components' parameters are listed there.

## 3. Optimal Control

For every second, *P*_*p*_in_(*t*) is supposed to be known, and with the shaft speed *w*_*p*_(*t*) as feedback, the required torque *τ*_*p*_(*t*) could be calculated. Thus, in all, these variables are considered as inputs to the power-train system. Then, the optimal control problem could be addressed in a mathematical way like the following:

(4) min⁡ J=∑t=0t=τ( ∫ττ+1m˙fuel(theng(t),τeng(t))dt  +f(SOC(t)∫ττ+1m˙bat(t)dt)),m˙bat=τmcwmc/ηmckδ·ϑ.

Subject to:

(5) theng_min⁡≤theng(t)≤theng_max⁡,τeng_min⁡≤τeng(t)≤τeng_max⁡(weng(t)),wmc_min⁡≤wmc(t)≤wmc_max⁡,τmc_min⁡(wmc(t),SOC(t))  ≤τmc(t)≤τmcmax⁡(wmc(t),SOC(t)),SOCmin⁡≤SOC(t)≤SOCmax⁡,

where ${\dot{m}}_{\text{bat}}$ is equivalent fuel rate of the battery, *δ* is a gain to convert unit Ws to kWh, and *ϑ* is the average fuel rate of the engine. th_eng_min⁡_ and th_eng_max⁡_ are minimal and maximal throttle angle of the engine, *τ*_eng_min⁡_ and *τ*_eng_max⁡_ are minimal and maximal engine's torque, *w*_mc_min⁡_ and *w*_mc_max⁡_ are speed thresholds for the motor, and *τ*_mc_min⁡_ and *τ*_mc_max⁡_ are torque boundaries of the motor which are function of *w*_mc_ and SOC. SOC_min⁡_ and SOC_max⁡_ are limitations for battery's SOC.

 The previous formulation is based on the concept of equivalent fuel consumption, and the objective is to find the global optimum by searching the entire possible space. The engine's fuel rate is indexed by the throttle angle and torque of the engine. For any given required power at each one-second period, engine's different working points will bring about different amounts of fuel consumption. So here we define the throttle angle and torque as the input variables. For a single time interval, the optimum will be found with lowest equivalent fuel consumption. Then moving to the next time interval, so a set of optimal points would be achieved, and for every potential point, it is necessary to check its feasibility so as to meet all the constraints as shown in Table 2.

**Figure 3:**
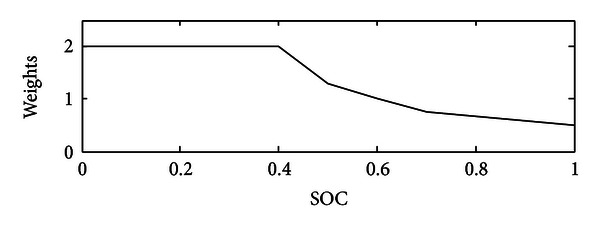
SOC weighting factor *f*(SOC) for optimal control.

 In objective function, weight is introduced, that is, function of battery's SOC, to maintain SOC in narrow range (see Figure 3). Once SOC is 0.6, the weight is equal to 1, and when SOC increases, the weight would be slightly changed to 0.5. Furthermore, if SOC changes in the opposite direction, our program will finally increase the weight to 2 that would bring about impacts of using battery less and operating engine in high output power level. 

**Figure 4:**
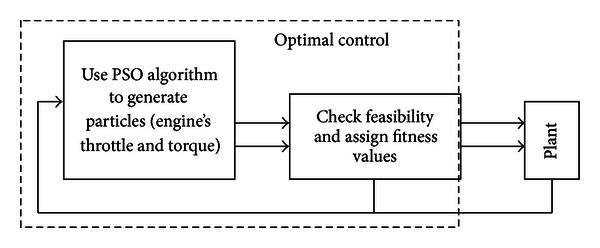
Structure of the optimal control.

 As described in Figure 4, the plant block will give the required torque demand to the PSO block (the required power demand at the pumps); then PSO will initialize a group of particles with random positions and velocities that are located in the predefined ranges. Here, our space is defined by engine's throttle angle and torque, and the outputs of PSO block are commands for the engine and motor; then these commands will be delivered to the checking feasibility block, and if only one command in the set does not satisfy the constraints, a relative high value will be assigned to the corresponding fitness function. In other words, these unfeasible points will not been selected to generate next population. After the first generation is totally completed, PSO uses the following two equations to reproduce next points:

(6) vdi(k+1)=w(k)vdi(k)+c1·rand·(pBesti(k)−xdi(k))+c2·rand·(gBest(k)−xdi(k))xdi(k+1)=xdi(k)+vdi(k+1),

where *i*, *d*, and *k* represent the number of particles, dimensions of each particle, and iterations, respectively. *v* and *x* are the velocity and position of each particle, and *p*Best^*i*^(*k*) indicates after *k* iterations, the position of one point with best fitness value located in number *i* particles. So this is a local minimum which is obtained only by one group of particles. *g*Best(*k*) means, after *k* iterations, the position of one point with best fitness value for all the particles, so this is a global minimum. Obviously, after a relatively large number of iterations *p*Best^*i*^(*k*) and *g*Best(*k*) will approach to the same position. 

**Figure 5:**
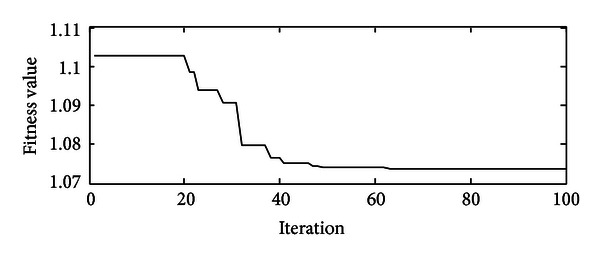
Best values for each iteration.

 As listed in Table 3, all the parameters related to the PSO have been defined. Here, the iteration *k* is a stopping criterion. We need to find out how many generations are needed for each optimization process to finally reach the optimum point. One case of optimization process is then launched in order to observe how the global best fitness value evolves when the number of iteration is increasing. In Figure 5, when iteration arrives at 80, it seems that the improvement will not be easily achieved. So we would state that the optimization process will approach its optimum after 80 iterations, and it is reasonable to set 80 iterations as the stopping criterion in PSO. 

**Figure 6:**
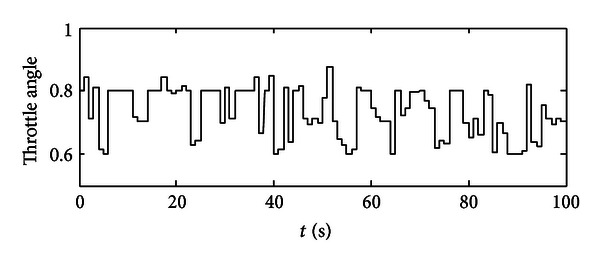
A group of optimized throttle angle demands for the engine.

**Figure 7:**
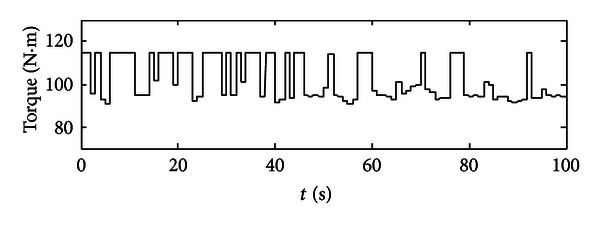
A group of optimized torque demands for the engine.

After each time interval, the optimum set of engine's throttle angle and torque could be recorded as well as the SOC value at the end of this time interval. So for the next time interval, the previous SOC value will be used as the initial SOC value. After 100 seconds, we could get a series of optimum points which are optimized for the given working condition in the specific time periods (see Figures 6 and 7). In Figure 6, the optimum throttle angle is plotted, since we get the optimum results in a period of one second, and also this is command signal, so one throttle angle value will last at 1 second and will be changed to another value steeply. In Figure 7, the optimum engine torque is located approximately from 85 to 115 N·m, and the engine will deliver the average required torque to the final load. Then, these two groups of optimum points will be used as control commands in model-based study. Some extensions would then be obtained. 

**Figure 8:**
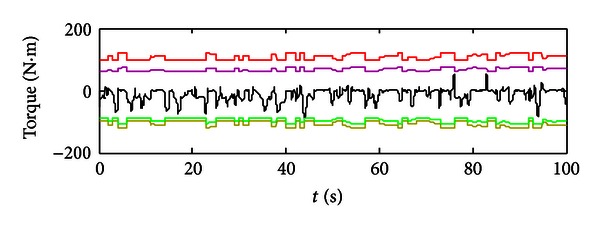
The motor's performance using optimum engine's points as commands.

**Figure 9:**
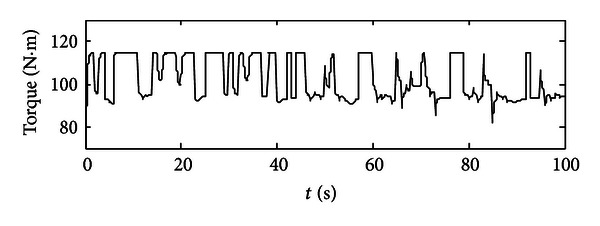
The engine's real-time torque along 100-second period.

**Figure 10:**
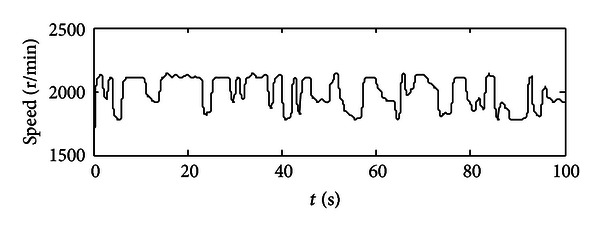
The engine's real-time speed along 100-second period.

In Figures 8–10, these are results got from using the aforementioned optimum points of the engine.  Figure 8 depicts the motor's performance in terms of its real-time torque (black curve), maximum propelling torque (red curve), maximum regenerating torque (yellow curve), and battery's constrains on the motor (pink curve and green curve). So we could get a conclusion that the motor is operated properly in reasonable range that meets all the constraints. In Figure 9, the engine's torque is plotted; since in engine's model, engine's dynamic characteristics (e.g., engine's response time) are taken into consideration, so the engine's real-time torque does have some difference from the engine's torque commands. Also we could calculate the real-time engine's speed which is equal to the motor's speed and pumps' speed using the engine's speed tuning characteristic curve.

**Figure 11:**
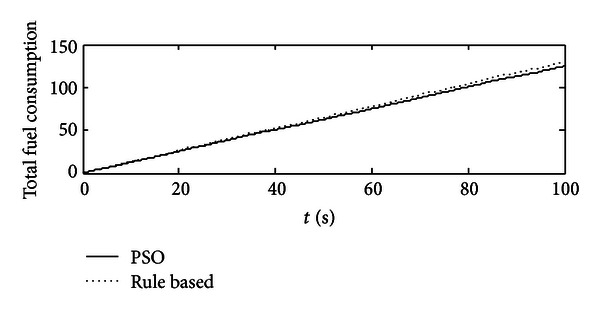
The comparisons of total fuel consumption between PSO optimal control and rule-based one.

**Figure 12:**
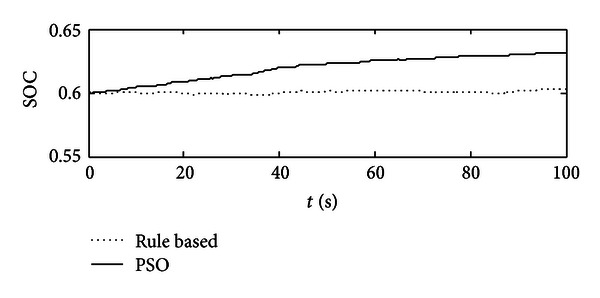
The comparisons of SOC alteration between PSO optimal control and rule-based one.

In Figures 11 and 12, the comparisons are studied. For these two control strategies, under the same circumstance, that is, the same initial SOC value, same system configuration, same working cycle, and so on, we would compare their results, especially in terms of fuel economy and system efficiency. In Figure 11, compared with rule-based one, a slight fuel economy could be achieved, but not too much, because at that point, the engine is operated constantly with 80% throttle angle which is suitable for that working cycle once using rule-based controller, but the reality is that the working condition for excavators is very bad, and the rule-based one would not fit for all the conditions. In addition, rule-based control will have the drawbacks as follows: (1) it selects SOC as its signal to decide engine's output power level, and compared with load alteration, SOC value is supposed to have some delays; (2) during the transitions of different working cycles, it cannot have high-level time response. In Figure 12, SOC value is increased more if using optimal control, which means that the battery is receiving much more energy from the engine, but referring to Figure 5, the throttle angle of the optimal control is obviously lighter than that of the rule-based one, and it indicates that the optimal control decreases the throttle angle for the engine but increases the efficiency of the system. 

## 4. Conclusion

 In this paper, the optimal control problem in parallel hybrid hydraulic excavators is first brought forward on the basis of analysis of components' dynamic in power-train and parameters. One may reach a conclusion that higher fuel economy would be achieved easily with hybrid configuration, but cannot imagine how much the system's efficiency and fuel economy would be increased with hybrids or exactly potential room for using this configuration. The optimal control results would give a benchmark in this field, so other control strategies could compare their effects with the previously stated standard one, and it has been clearly demonstrated that parallel hybrid system with optimal control is able to increase the fuel economy compared with rule-based control strategy in all kinds of working cycles. This comparison would give us a right direction in helping redesign components' size and revise control strategies. 

The current work is done only under heavy digging working condition, so the results of rule-based control strategy are not that bad, but extending to the blended working conditions, the rule-based one would never change its throttle angle quickly enough, so we will see a large difference between these two strategies. For further research work, there at least exist two aspects which could be studied deeply to update our results. One is to focus on the blended working conditions, trying to find rules on how to adjust the engine's throttle angle to get high fuel economy. Finally, to develop real-time application for PHHE that would become available. The other one is to use the optimal control results as guidance to revise rule-based control strategy accordingly.

## Figures and Tables

**Table 1:** Components' parameters of a PHHE.

Components' parameters	Specific information
Engine	
Type	ZN485Q, turbocharged diesel ICE
Rating power	25 kW at 2200 r/min
Maximum torque	124.8 N·m at 1860 r/min
Motor	
Type	Custom-built PMSM
Rating power	15.7 kW
Maximum torque	150 N·m
Rating voltage	250 V
Voltage range	216~360 V
Speed range	0~4000 r/min
Battery	
Type	NIMH, QNFG 8
Each cell's voltage	1.2 V
Number of cells	200
Energy capacity	8 Ah
Maximum discharge power	19.2 kW
Hydraulic pumps	
Type	2 variable pumps and 1 gear pump
Maximum flow	(2∗44 + 32) L/min
Working pressure	21 MPa

**Table 2:** Constraints parameter in optimal control formulation.

Constraints parameter	Value
th_eng_min⁡_~th_eng_max⁡_	0.5~1
*τ*_eng_min⁡_ ~*τ*_eng_max⁡_	0~124.8 N·m
*w*_mc_min⁡_ ~*w*_mc_max⁡_	−4000~4000 r/min
*τ*_mc_min⁡_ ~*τ*_mc_max⁡_	Subject to battery's SOC and motor's current speed
SOC_min⁡_~SOC_max⁡_	0.2~0.92
*ϑ*	240 g/kWh
*δ*	2.78 × 10^−7^

**Table 3:** Parameters in PSO.

PSO parameters	Value
*w*(*k*)	0.9*~*0.4, with a reduction of 1/120 for each *k*
*c*_1_	1.4
*c*_2_	1.4
*d*	2, throttle angle: 0.5~1; engine torque: 0~124.8 N·m
*i*	20
*k*	80
